# Riparian woody plant communities in the Romanian Carpathians: Species diversity and community structure of *Salix* and *Hippophaë* communities

**DOI:** 10.1002/ece3.11361

**Published:** 2024-05-21

**Authors:** Claudia Bita‐Nicolae, Larisa I. Florescu, Dorina Purice, Ozkan Kaya

**Affiliations:** ^1^ Department of Ecology & Nature Conservation, Institute of Biology Bucharest Romanian Academy Bucharest Romania; ^2^ Erzincan Horticultural Research Institute Republic of Türkiye Ministry of Agriculture and Forestry Erzincan Turkey; ^3^ Department of Plant Sciences North Dakota State University Fargo North Dakota USA

**Keywords:** conservation, habitat, *Hippophaë* community, riparian vegetation, *Salix* community, woody plants

## Abstract

Riparian woody plant communities, including shrubs and trees, are essential for maintaining biodiversity, protecting against floods, reducing erosion, and transporting nutrients. However, these habitats are greatly threatened by human activities, particularly agricultural land acquisition, and the introduction of invasive species. This study examined species diversity and interspecific association in riparian woody plant communities along rivers in the Romanian Carpathians. The study focused on communities of *Salix purpurea, S. alba*, and *Hippophaë rhamnoides* in mountain regions, with varying sampling efforts at different sites for each species. A total of 174 plant species were found, predominantly herbaceous (77.9%), followed by trees (11.6%) and shrubs (10.5%). While *S. alba* and *S. purpurea* communities show high species richness and abundance, *S. alba* has slightly higher diversity (*H*′ ≈ 2.23, SD = 0.28) than *S. purpurea* (*H*′ ≈ 1.69, SD = 0.42). Contrarily, significant differences exist between *H. rhamnoides* and *S. alba* communities in species richness (*p* = .007) and Shannon diversity (*p* = .004). PCA analysis elaborated on distinct distribution patterns of plant associations within habitats *S. purpurea* community, *H. rhamnoides* community, and *S. alba* community. Four invasive species (*Oenothera biennis* L. and *Oxalis stricta* L. in *S. alba* communities, *Reynoutria sachalinensis* Nakai in both *S. purpurea* and *H. rhamnoides* communities, and *Erigeron canadensis* L. in *H. rhamnoides* communities) were identified, as requiring conservation efforts. Hemicryptophytes dominate species richness, while microphanerophytes and megaphanerophytes significantly contribute to plant abundance. *H. rhamnoides* formed *Hippophaë rhamnoides* dunes (2160) Natura 2000 habitat, while *S. alba* created galleries within the 92A0 *Salix alba* and *Populus alba* habitat. In conclusion, the findings from this study highlight the importance of preserving riparian habitats because their value goes beyond local or regional considerations and extends to the global scale due to their unique characteristics.

## Graphical abstract

This article examines riparian woody plant communities in the Romanian Carpathians, focusing on *Salix purpurea*, *S. alba*, and *Hippophaë rhamnoides* communities in mountain regions. The study reveals a total of 174 plant species, with herbaceous species being the most abundant. *Salix alba* and *S. purpurea* communities exhibit high species richness and abundance, while *H. rhamnoides* communities have distinct community structures characterized by low evenness and dominance of certain species. The results highlight the importance of protecting these habitats, which are classified as galleries 92A0 *Salix alba* and *Populus alba*, and 2160 Dune with *Hippophaë rhamnoides* under the Habitats Directive in Romania.

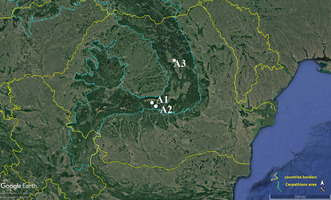

## 1 INTRODUCTION

The riparian zone is the area where land and a river or watercourse meet (Rivas‐Martinez, 1982; Schmidt et al., 1991). It is special in that it combines characteristics of terrestrial and aquatic ecosystems (Naiman et al., 1993) and is known for its high biodiversity. Hydrological and geomorphological variations in riverbanks lead to a diversity of structural and functional features in the riparian zone (Décamps, 1996; Vesipa et al., 2017), which play an important role in maintaining the biodiversity of the watershed and the surrounding landscape (Capon & Pettit, 2018; Naiman & Décamps, 1997). The presence of plant communities is relevant in defining river and stream characteristics (Osborne & Kovacic, 1993), and riparian zone structure is determined by the continuity of vegetation along the river, the width of the naturally vegetated channel, and the diversity of plant species and their arrangement (Beschta & Ripple, 2012; Dala‐Corte et al., 2020; Fonseca et al., 2021; Tánago & Jalón, 2006). While some *Salix* species, such as *S. alba* and *S. purpurea*, are strong components of riparian forests and occur in early‐successional sites, including post‐mining areas with low water levels, others in this genus, such as *S. triandra*, also colonize these sites. While *Salix* species usually demonstrate a significant connection between their hydrological and climatic needs, this suggests that these species thrive in favorable environmental conditions, however, may have difficulty adapting to drought or local water level fluctuations (Butterfield et al., 2021). Furthermore, these species tend to be more widespread in areas with higher summer rainfall and are often found further away from watercourses (Cavender‐Bares et al., 2004). These distinctions indicate the varying ecological roles of these *Salix* species within the riparian landscape. By analogy, *H. rhamnoides* positively impacts overall community structure, biodiversity, and soil quality (Zhang & Chen, 2007). It also plays a role in maintaining the nutrient balance of the ecosystem by adding nitrogen and decomposing plant debris (Ruan & Li, 2002). It has, however, been noted that both these species and other species adapted to riparian areas significantly affect the physical and chemical properties of streams (Hickin, 1984). Indeed, these species create habitats and provide nourishment for both land and water‐based organisms (Fierro et al., 2017; Lind et al., 2019). It is also known that the vegetation along rivers serves various purposes, including reducing erosion, filtering pollutants, and providing shelter and food for animals (Bennett et al., 2014), as well as helping replenish soil moisture by retaining summer rains when groundwater is scarce (Johnson & Almlöf, 2020).

Riparian forests, which have historically been crucial for human settlements (Ferreira et al., 2005) are under long‐term pressure from deforestation due to their proximity to early human habitations near rivers. Human activities altering the structure of riparian vegetation have immediate and direct effects on primary consumers by decreasing their access to food, water, and breeding and nesting sites (Dala‐Corte et al., 2020; Fierro et al., 2017; Forio et al., 2020). This also decreases the amount of organic matter available as a nutrient source for aquatic ecosystems and reduces the ability of the area to buffer and retain floodwaters (Cavender‐Bares et al., 2004; Forio et al., 2020; Hoppenreijs et al., 2022). Human activities, such as urbanization and agriculture, can lead to habitat destruction and pollution, while climate change affects temperature and precipitation. These combined factors can alter riparian vegetation, impacting biodiversity and resilience (Beschta & Ripple, 2012; Capon & Pettit, 2018; Décamps, 1996; Naiman et al., 1993; Naiman & Décamps, 1997; Schmidt et al., 1991; Tánago & Jalón, 2006; Vesipa et al., 2017). Numerous studies have demonstrated the profound significance of riparian zones. The primary focus here is on the broader goal of conserving these critical ecosystems. This emphasis on conservation drives the examination of vegetation composition, structure, and protection in this context (Butterfield et al., 2021; Dala‐Corte et al., 2020; Fonseca et al., 2021).

Raunkiaer's system (Raunkiaer, 1934) is considered a very useful and practical tool for the ecological classification of plant communities in the use of life forms to compare plant assemblages in plant community ecology (Giménez, 2004; Mota, Watanabe, et al., 2018). While many functional classifications exist, Raunkiaer's life forms are the most widely used due to their simplicity and effectiveness. The system groups plants into five major categories based on their protection strategies during unfavorable seasons and the height of their renewable buds relative to the soil surface. These characteristics reflect different tolerances toward climatic variables and can help in investigating evolutionary processes that shape species assemblages and their morphological traits (Bhattarai & Vetaas, 2003; Giménez, 2004; Mota, Watanabe, et al., 2018).

Regarding previous studies, riparian forests and vegetation in montane and submontane belts are studied in Europe (Guarino et al., 2008; Milanovi & Stupar, 2017; Schwabe, 1989), and in the Western Carpathians, as well (Sienkiewicz et al., 2001; Slezák et al., 2020). The Romanian Carpathians are also a well‐researched area in terms of systematic vegetation and flora, with the studies previously carried out in locations in our study area (Beldie, 1967; Biţă‐Nicolae, 2011).

Several studies have explored plant species distribution and life forms in diverse geographic and climatic conditions. Di Biase et al. (2021) identified hemicryptophytes and chamaephytes as well suited for extreme conditions like low temperatures and drought. Irl et al. (2020) applied Raunkiær's plant life form concept to subtropical islands, revealing distinct patterns in geography and climate. There is also a national list of life forms for wild species in Romania, documented by Biță‐Nicolae and Sanda in 2011. Nonetheless, the specific ecological and botanical characteristics of the riparian vegetation in the Romanian Carpathians remain underexplored. This study aims to provide a comprehensive analysis of these habitats, addressing various important issues such as their role as biodiversity hotspots, provision of ecosystem services, impact on climate change, conservation priorities, and filling gaps in scientific knowledge. Given the limited knowledge on riparian shrubs in the Romanian Carpathians, especially those of conservation importance (Bita‐Nicolae, 2023), it is imperative to establish a clear understanding of the subject. This study aims (I) to identify the structural characteristics of these communities, including species composition, diversity, and relative abundance; (II) to assess the conservation status of *S. purpurea*, *S. alba*, and *H. rhamnoides*; (III) to elucidate the similarities and distinctions between them, highlighting the inherent heterogeneity of riparian shrublands as ecological entities and (IV) consider carefully the classification of these communities into distinct habitat types.

By approaching these objectives, our research aims to complete a knowledge gap in riparian ecology within the Romanian Carpathians. Understanding the diversity and structure of riparian forests is important for effective conservation efforts and sustainable management practices. This study not only provides valuable information about these ecosystems but also provides a basis for informed decision‐making and policy development, ultimately improving the conservation and management of riparian shrublands in this ecologically significant region.

## 2 MATERIALS AND METHODS

### 2.1 Area of study and sampling sites

**FIGURE 1:**
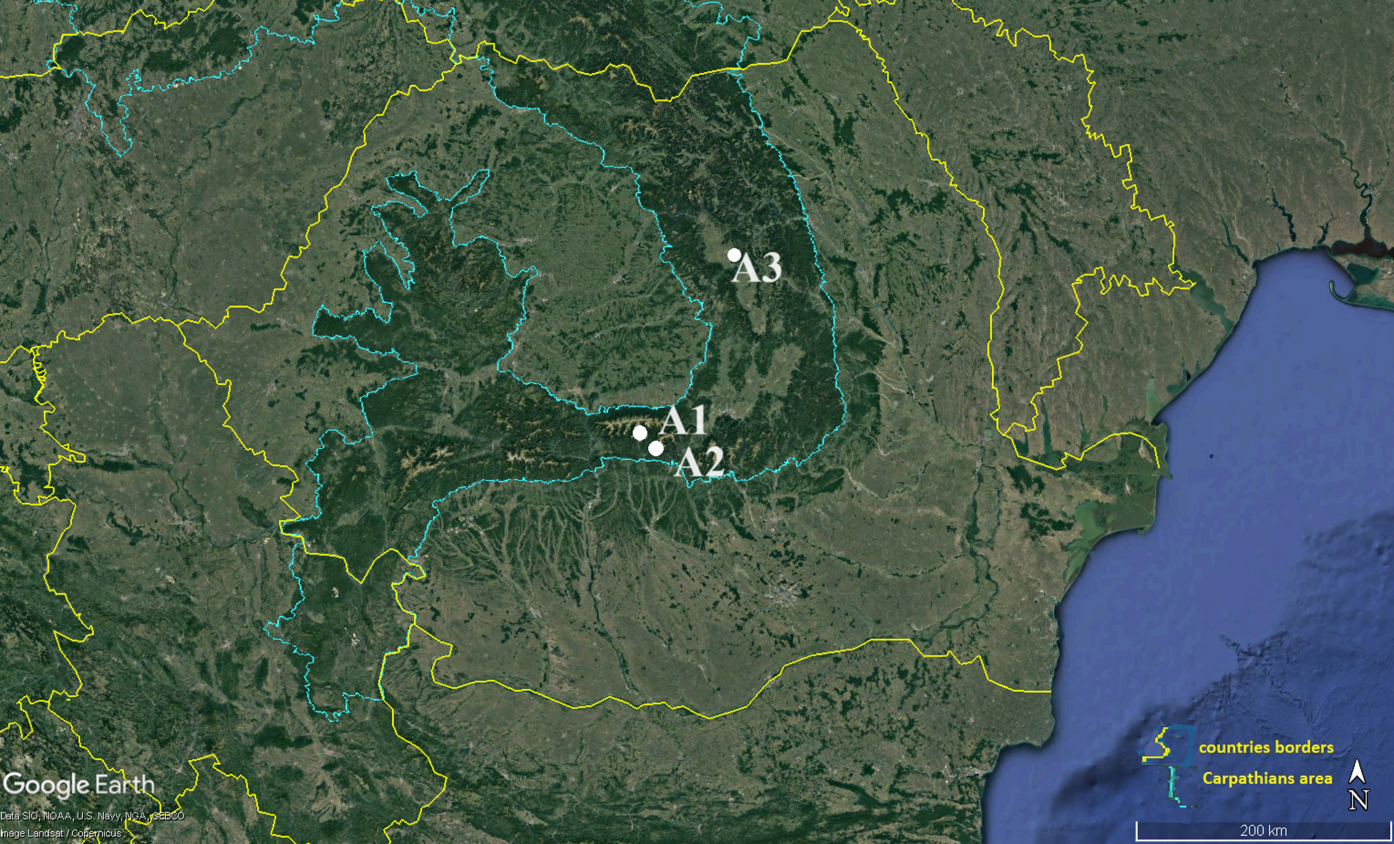
The Carpathian and the studied sites (modified from Google Earth).

The Carpathians (Figure 1) are Europe's largest, longest, most sinuous, and fragmented mountain chain (Bălteanu, 2012). The Carpathian Mountains are particularly interesting because of their both wilderness and biodiversity (United Nations Environment Programme, 2007). The entire Carpathian arc (47.2390° N, 25.5909° E) stretches across Central and Eastern Europe. They are the second longest mountain range in Europe with a length of about 1.500 km. In this context, the Romanian Carpathians is the longest sector of the Carpathians (910 km corresponding to 54% of the total length of the Carpathians) (United Nations Environment Programme, 2007). The most common rock in the Carpathians is flysch (Bălteanu, 2012). The climate is characteristic of mountainous‐subalpine regions; the location and rugged relief strongly influence the distribution and unilateral or multiple influences of general climatic or microclimatic factors (Micu et al., 2016). At medium altitudes (1000 to 1500 m), temperatures range from 4°C to 8°C (39°F to 46°F) and rainfall is about 1100 mm per year (Micu et al., 2016).

The Carpathian Mountains region is known for the valuable ecosystem goods and services it provides, such as food, fresh water, forest products, and tourism (Turnock, 2008). An important ecosystem found in the Carpathians is wetlands, which not only play a crucial role in biodiversity conservation but also provide a range of ecosystem services essential for human well‐being (Gałka et al., 2020). These habitats include riparian vegetation, aquatic habitats, wet meadows, peatlands, wet forests, watercourses, and subterranean wetlands (Romanescu et al., 2011). Prudent, however, is the recognition of the need for further comprehensive research to validate these findings. Additionally, the vegetation studied is prevalent across the entire Carpathian chain (Bita‐Nicolae, 2022), enhancing the potential relevance of the study's findings. Slope and exposure characteristics were recorded for every plot within each studied community (Table 1). These attributes serve as fundamental factors influencing the ecological dynamics and species distribution within the Carpathian Mountains. The detailed documentation of slope gradients and aspects provides crucial insights into the local environmental conditions shaping the biodiversity and ecosystem dynamics of the region.

**TABLE 1:** Sites parameters.

Community of	*Salix alba* L.	*Hippophaë rhamnoides* L.	*Salix purpurea* L.
Altitude (m)	300–950	500–580	420–550
Slope (degrees)	25–40	15–25	25–55
Area of sampled plots (m^2^)	50, 100	100	25, 50, 100
Total vegetation cover (%)	40–90	65–100	65–100

### 2.2 Field methods

The study investigated plant communities of three different species, namely *Salix purpurea* community, *Hippophae rhamnoides*, and *S. alba* to assess species richness and composition. The *S. alba* community was sampled at 10 different sites (A30–A39), with a total of 101 relevés taken. The *H. rhamnoides* community was studied at 6 sites (A21–A26), with a total of 67 relevés taken. The *S. purpurea* community was sampled at 5 sites (A11–A15), with a total of 25 relevés taken. The difference in the number of recorded relevés for each plant community is due to variations in the size and complexity of each community. Thus, larger and more diverse communities, such as *S. alba*, required a greater number of surveys to adequately capture species richness and composition. We recorded plots of 50 m^2^ and 100 m^2^ for *S. alba* communities, 100 m^2^ for *H. rhamnoides* communities, and 25, 50, and 100 m^2^ for *S. purpurea* communities (Table 1). The variations in plot size, or area, are determined on a case‐by‐case basis, taking into account the total area of the community's location and the challenges associated with accessing specific land areas. The selection of diverse plot sizes was a strategic choice based on the unique attributes of each syntaxon and logistical constraints. However, interpreting our findings while considering the potential biases arising from these differences in event plot dimensions indicates the need for careful, emphasizing the need for cautious interpretation and acknowledging the limitations in the broader applicability of our results.

This study was carried out in a group of river catchments located in the Romanian Carpathians, in the mountain belt. The sites are located in natural areas belonging to the Prahova and Bistrita rivers of the Romanian Carpathians. The study was conducted in the spring and early summer (April to June), which are the months with maximum vegetation, in 2015, 2017, and 2020.

In our phytosociological surveys, we applied the Braun‐Blanquet phytosociological method (Braun‐Blanquet, 1964), following the guidelines established by Braun‐Blanquet in 1964. This method traditionally involves preferential sampling of vegetation units, but we adapted it to ensure the robustness and representativeness of our data. The guiding principle for this selection was to cover a single vegetation type, maintaining a more balanced representation of the community. Central to the Braun‐Blanquet method is the use of relevés, which are standardized plots in which all plants within a defined area are meticulously recorded and identified. By employing this approach, we effectively alleviated potential biases, ensuring comparability of data across vegetation communities and thereby enhancing the reliability of our research results. The recording and identification within relevés minimized the risk of overlooking or misclassifying plant species, contributing to a more comprehensive and unbiased dataset. Taxonomy of plant species follows Euro+MedPlantBase (ww2.bgbm.org/EuroPlusMed) and The Plant List (www.theplantlist.org). We used national literature, as well (Biță‐Nicolae & Sanda, 2011). Moreover, for each relevés, we recorded the following data: locality, altitude (m a.s.l.), slope aspect (degrees), plot area (m.sq.), total vegetation cover (%) (Table 1), and cover‐abundance values of the species, the latter assigned using the Braun‐Blanquet scale (Braun‐Blanquet, 1964). The vegetation classification followed the EuroVegChecklist (Mucina et al., 2016), a widely utilized standardized reference system in phytosociology for syntax classification. According to Mucina et al. (2016), the three studied communities fall under the *Salicetea purpureae* Moor 1958 class, specifically within the order *Salicetalia purpureae* Moor 1958. However, the communities featuring *S. purpurea* and *H. rhamnoides* belong to the alliance *Salicion eleagno‐daphnoidis* (Moor 1958) Grass 1993. These communities represent low open forests consisting of willow and poplar trees, typically found in lowland to submontane alluvial river areas within the immoral zone of Europe. On the other hand, the community with *S. alba* belongs to the alliance *Salicion albae* Soo 1951, characterized as willow and poplar low open forests within lowland to submontane river alluvial regions in the nemoral zone of Europe. To simplify the analysis for many surveys, we used a synoptic table with constancy values that were coded in percentages (Table S1). The use of synoptic tables allowed for the use of our material that presented only simplified frequency class values. We recorded the categories as follows:

Class V: 81%–100%.
Class IV: 61%–80%.
Class III: 41%–60%.
Class II: 21%–40%.
Class I: 1%–20%.

The percentages represent ranges for each category.

### 2.3 Phytosociological framework

All communities studied belong to the *Salicetea purpureae* Moor 1958 class, specifically encompassing willow and tamarisk scrub, along with low open forests in riparian habitats spanning the temperate to arctic zones of Europe (Mucina et al., 2016). Our analysis focused on each community from a phytosociological standpoint, all of which have been classified by Mucina et al. (2016) within the Central and Eastern European Group of Alliances.

The S. *purpurea* community is a community of species of willow commonly found growing in strips along rivers in hilly regions, which helps stabilize soil and prevent erosion (Iwata et al., 2003). As noted by Schickhoff (2005), these willow species predominantly thrive in the middle and northern boreal regions, particularly in pioneer habitats such as stream and river margins. We conclude that this community aligns with the *Salicetum purpurea* association (Soó, 1934) Wendelbg‐Zelinka 1952 (Sanda et al., 2008), observed not only in Central Europe but also in the Western Carpathians (Douda, 2016; Silc, 2003; Towpasz et al., 2008). The dominant species observed in our study closely resemble those reported previously, including a mix of meadow and ruderal species like *Equisetum arvense, Mentha spicata, Ranunculus repens, Solanum dulcamara*, and *Urtica dioica* (Douda, 2016; Silc, 2003; Towpasz et al., 2008). Additionally, in the absence of significant disturbance, succession within this community may lead to its replacement by *Alnus incana* woodland (Douda, 2016).

The *H. rhamnoides* community is commonly found in areas previously deforested, such as eroded surfaces, landslides, and clay or marl substrates (Oriolo & Poldini, 2002; Sburlino, 2020). Correspondingly, the association for this studied community is the *Salici incanae‐Hippophaetum* Br‐Bl. (Volk 1939) (Sanda et al., 2008). As per Mucina et al. (2016), the order is *Salicion eleagno‐daphnoidis* (Moor 1958) Grass 1993, signifying willow scrub on gravelly stream banks in the submontane to subalpine belts of the Alps, Pyrenees, and Carpathians.

The *S. alba* community is commonly found along rivers and streams, forming narrow strips adjacent to water bodies at altitudes up to 335 meters (Neuhäuslová & Douda, 2013). This community aligns with the *Salicetum albae* Issler 1926 association. Dominated by *S. alba*, often accompanied by varying dominance levels of *S. fragilis* (Neuhäuslová & Douda, 2013; Triest et al., 2000), this community's plant species are adapted to wet and waterlogged conditions. Species like *Populus alba, Clematis vitalba, Calystegia sepium, S. triandra, Vitis sylvestris, Cucubalus baccifer*, and *Eupatorium cannabinum* are commonly found here. In most sites, this vegetation exhibits a high invasion potential, frequently accompanied by invasive non‐native plants like *Solidago canadensis, Reynoutria japonica*, and *Helianthus tuberosus* (Wagner et al., 2017). However, our observed sites remain relatively free from such invasions.

### 2.4 Data analysis

The data resulted after the Braun‐Blanquet quantitative method was used in the statistical analyses without being transformed. The analysis considered the characteristics of the existing populations, including the number and abundances of species. The characteristics of the present populations in terms of the number of species and abundances were thus used in further analysis. Data were statistically processed using Past software and XLSTAT pro (Florescu et al., 2022; Hammer et al., 2001; XLSTAT pro, 2013).

The field data were used in the estimation of diversity indices Species richness (S), Shannon's index (*H*′), Evenness index – (*eH*/S), and the Rényi diversity. The diversity indices used are important indicators for evaluating the ecosystems.

$${\text{Shannon}}^{’}s\;\text{index}\;\left(H^{\prime }\right)=\sum \mathrm{pi}\times \ln\left(\mathrm{pi}\right)$$

Calculated based on ∑: A Greek symbol that means “sum”; ln: natural log; *pi*: the proportion of the entire community made up of species *i*.

A higher value of *H*′ indicates a more diverse community, while a lower value indicates less diversity (Hammer, 2012).

Evenness index – (*eH*/*S*) – The uniformity of individuals of each species of the community. Evenness index was used to assess the distribution of species in a community or ecosystem. The index considers both the number of species and their relative abundance to determine how evenly individuals are distributed among different species. Evenness eH/S index or Buzas and Gibson's evenness is used to quantify the evenness of species distribution within a community.

It is calculated by the formula

$$e^{H}/S=\frac{H^{\prime }}{\ln\left(S\right)}$$

where *H*′, the Shannon diversity index; *S*, total number of species.

The index ranges from 0 to 1, where 1 means a completely even distribution, suggesting a balanced ecosystem with no dominant species. On the other hand, a low Evenness index suggests that one or a few species dominate the community, leading to an uneven distribution (Hammer, 2012).

The Rényi diversity profile is an ordering method and useful for comparison between different sites providing an overview by generating curves that include Species richness (*α* = 0), Shannon‐Wiener diversity index (*α* → 1), Simpson index (*α* = 2), and Berger‐Parker (*α* → ∞). The compositional complexity of an ecosystem and comparison of assemblages are suitable for the diversity profiles. Rényi diversity curves were used to highlight the profiles of the three areas from the point of view of diversity indices. The analysis evaluates the distribution of species abundance within a community and compares the diversity of different communities. The method is also known as Rényi entropy and is calculated based on the species present and their relative abundance. The higher diversity is represented by the higher profile curve (Chao & Jost, 2015; Guo et al., 2013; Hammer, 2012; Wafa'a, 2022).

Diversity indices were calculated separately for each survey and the Kruskal–Wallis analysis of variance test was employed to assess differences in species richness and Shannon diversity among the relèves in areas *Salix purpurea* community (A1), *H. rhamnoides* (A2) community, and *S. alba* community (A3). The Kruskal‐Wallis test is used to compare three or more groups. It is the non‐parametric equivalent of the Anova test, on the same principle of comparing medians between them. The method is used when the data violates the assumptions of parametric tests. Its null hypothesis assumes equal population means in all groups, the alternative suggesting that at least one group differs in mean. Dunn's post hoc test was applied to identify specific groups that differed significantly in the analysis. Dunn's procedure uses statistics based on the Z distribution, comparing rank differences between pairs of groups to determine significance (Dinno, 2015). For the multiple pairwise comparisons, a Bonferroni correction was used. The study aimed to analyze how riparian woody plant species were associated within the communities of the three investigated areas using Principal Component Analysis (PCA).

## 3 RESULTS

### 3.1 Field results

By taking multiple relevés across different sites, we were able to create a more comprehensive picture of the plant communities. Overall, the study found a total of 174 plant species across the communities studied. Most of the species were herbaceous (77.9%), followed by trees (11.6%) and shrubs (10.5%). The most common plant families were angiosperms, specifically Asteraceae (25%), Lamiaceae (17%), and Poaceae (15%). Three gymnosperm families were also found: Equisetaceae (2%), Pinaceae (2%), and Cupressaceae (1%). A synoptic phytosociological table was presented to provide a summary of the primary features of each plant community (Table S1).

### 3.2 Statistical results

**FIGURE 2:**
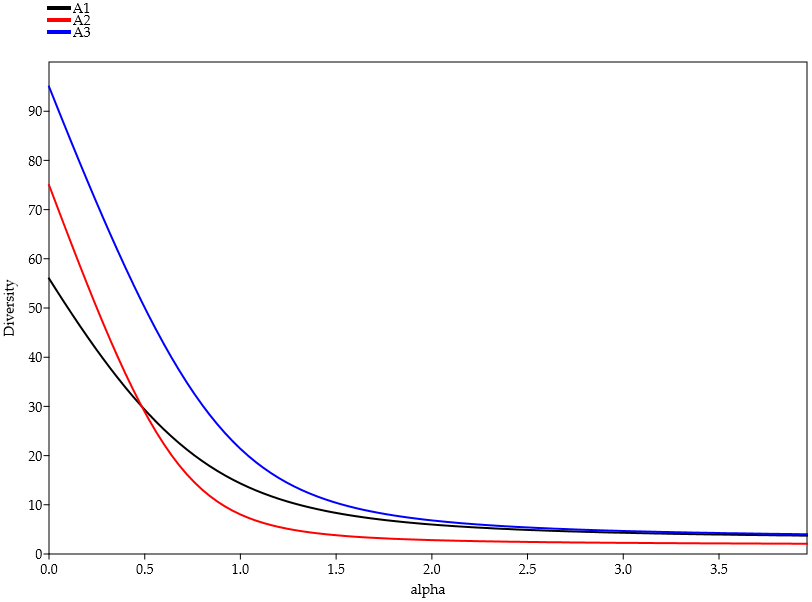
Renyi diversity profiles of the studied vegetation communities: *Salix purpurea* communities (A1), *Hippophaë rhamnoides* communities (A2), and *Salix alba* communities (A3) communities.

**FIGURE 3:**
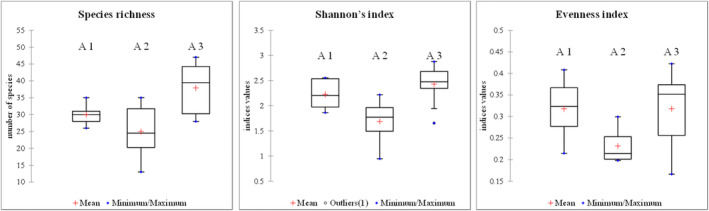
Diversity indices (S‐ species richness; Shannon's diversity index – *H′*, and Evenness‐E) evaluated for *Salix purpurea* communities (A1), *Hippophaë rhamnoides* communities (A2), and *Salix alba* communities (A3) communities. The boxplots contain the mean (red cross), and median (inside box line), the bottom of the boxes indicates the 25th percentile and the top of the boxes represents the 75th percentile, the T bars presenting the minimum and maximum values, with short horizontal lines “whisker.” “Outliers” are shown as circles highlighting the points extremely far from the other data.

Although the Rényi diversity profiles of the three areas (Figure 2), the trends of the studied communities were highlighted (Figure 3). At *α* = 0, *S. alba* community (A3) and *H. rhamnoides* community (A2) showed high species richness. In the case of the *H. rhamnoides* community (A2), the curve showed a sudden drop in the Shannon‐Wiener diversity index (*α* → 1); indicating the presence of species with low abundances. From *α* = 2, *S. purpurea* community (A1) and *S. alba* community (A3) overlapped, which indicated a similarity regarding the dominance of some species. Instead, the area of *H. rhamnoides* community (A2) exhibited a lower diversity profile when compared to the other two areas, indicating a weaker overall diversity. Analyzing diversity indices at the plot level helped us delve deeper into the characteristics of the study sites.

**FIGURE 4:**
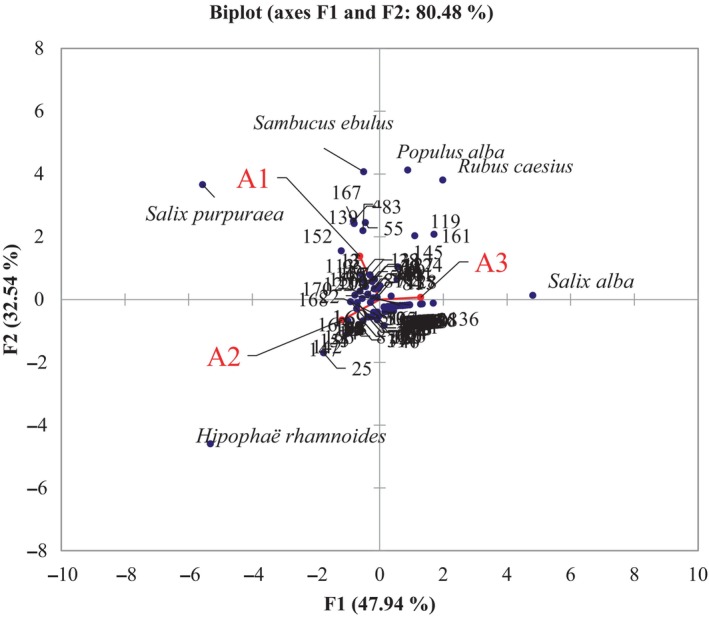
Principal component analysis based on species composition associated with the studied areas *S*. *purpurea* community (A1), *H. rhamnoides* community (A2), and *S. alba* community (A3). The full name of species used in the analysis are added to Table S1 by adding the number of each species into Table S1.

In terms of species richness, the area of *S. alba* community (A3) demonstrated the highest value of mean (37.9 ± 7.1), followed by *S. purpurea* community (A1) (30 ± 3.0), and *H. rhamnoides* community (A2) (25 ± 7.77) (Table 2). This richness was reflected in the Shannon diversity index as well, with *S. alba* community (A3) (2.43 ± 0.37) showing higher diversity compared to *S. purpurea* community (A1) (2.23 ± 0.28) and *H. rhamnoides* community (A2) (1.69 ± 0.42). Regarding evenness, all three areas exhibited relatively low values: *S. purpurea* community (A1) (0.32 ± 0.07), *H. rhamnoides* community (A2) (0.23 ± 0.04), and *S. alba* community (A3) (0.32 ± 0.09), indicating a similar distribution trend among species. Boxplot analysis (Figure 4) of the diversity indices highlighted significant differences between *H. rhamnoides* community (A2) and *S. alba* community (A3), which were further confirmed by the Kruskal‐Wallis test for species richness (*p*‐values of pairwise differences = .007) and Shannon diversity (*p*‐values of pairwise differences = .004). We mentioned significant results only.

**TABLE 2:** Descriptive statistics of the diversity indices in the study sites.

Statistic	*S* (A1)	*S* (A2)	*S* (A3)	*H*′ (A1)	*H*′ (A2)	*H*′ (A3)	*E* (A1)	*E* (A2)	*E* (A3)
Minimum	26.00	13.00	28.00	1.86	0.95	1.65	0.21	0.20	0.17
Maximum	35.00	35.00	47.00	2.55	2.22	2.88	0.41	0.30	0.42
Mean	30.00	25.00	37.90	2.23	1.69	2.43	0.32	0.23	0.32
Standard deviation (n)	3.03	7.77	7.11	0.28	0.42	0.37	0.07	0.04	0.09

*Note*: *E*, Buzas and Gibson's evenness; *H*′, Shannon's diversity index; *S*, species richness.

A biplot analysis is a powerful method used in multivariate statistics to visualize relationships between variables and observations in a data set. Biplot analysis was used to understand associations between plant communities *S. purpurea* (A1), *H. rhamnoides* community (A2), and *S. alba* community (A3). The results show that species composition remained highly divergent (Figure 4). Analyzing the PCA we find the cumulative value of the first two F1 + F2 axes exceeds 80%, suggesting a notable association between the studied variables, as depicted in Figure 5. Area *S. purpurea* (A1), representing a specific community, demonstrated a strong association with axis 2, evident from a coefficient of .79. Conversely, areas *H. rhamnoides* community (A2) and *S. alba* community (A3) were more closely linked to axis F1, displaying coefficients of .57 and .69, respectively. Notably, the *S. purpurea* A1 community was situated in a quadrant on the biplot opposite to the *H. rhamnoides* community (A2) association represented by *H. rhamnoides*, while the *S. alba* community (A3) was distinct from A2. This reaffirms the distinct distribution patterns of these three plant associations within their habitats.

Moreover, the biplot revealed a skewed representation of species, with only a few species appearing abundant, while the majority had lower weight in the analysis. *H. rhamnoides, S. alba*, and *S. purpurea* were identified as species significantly associated with characteristic areas. Furthermore, *Sambucus ebulus, Populus alba*, and *Rubus caesius* were also observed to have associations with both *Salix purpurea* community (A1) and *S. alba* community (A3) areas.

To better characterize the ecological context, data on the life forms of these species are summarized in Table 3. At all three sites, H (Hemicryptophytes are perennial, but their buds are located at or just below the soil surface) dominated in terms of species richness, but with low abundance (Table 3). The following species were highly represented as H: *Sambucus ebulus, Rubus caesius* (in *S. purpurea* community), *Eupatorium cannabinum* (in *H. rhamnoides* community), and *Rubus caesius* and *Urtica dioica* (in *S. alba* community). On the other hand, M (microphanerophytes, heathland shrubs or small trees) and MM (megaphanerophytes, trees) (plants with buds above the ground) species were few in the number of species but better represented in terms of abundance. For example, in *S. purpurea* community, species such as *S. purpurea, Populus alba*, and *Crategus monogyna* were mentioned with high abundance, while in *H. rhamnoides* community, *H. rhamnoides, S. purpurea*, and *Sorbus acuparia*, and in *S. alba* community, *S. alba*, and *Populus alba* were found to be more abundant.

**TABLE 3:** Raunkiaer life‐forms in studied sites: H hemicryptophytes, MM megaphanerophytes, M microphanerophytes, Th annual therophytes, G geophytes, TH biannual therophytes, Ch chamaerophytes, N nanophanerophytes, HH hemihydrophytes; A1 *S. purpurea* communities, *H. rhamnoides* communities (A2), and *S. alba* communities (A3).

Life‐forms no. sp./%	A1	A1%	A2	A2%	A3	A3%
H	34	60.71	36	48	39	41.05
MM	3	5.36	12	16	14	14.74
M	4	7.14	11	14.67	12	12.63
Th	4	7.14	7	9.33	10	10.53
G	4	7.14	1	1.33	9	9.47
TH	4	7.14	5	6.67	5	5.26
Ch	2	3.57	1	1.33	3	3.16
N	1	1.79	2	2.67	2	2.11
HH	0	0	0	0	1	1.05

Broadly, the results suggest that hemicryptophytes dominate in terms of species richness at the three sites, but M and MM contribute significantly to the abundance of plant species. Furthermore, these species tend to be more widespread in areas with higher summer rainfall and further away from watercourses.

## 4 DISCUSSION

The study of three different riparian communities, including *S. purpurea*, *H. rhamnoides*, and *S. alba*, revealed distinct patterns in terms of species diversity and distribution. Our results showed that a total of 174 plant species were identified in the communities, providing insight into the biodiversity of the ecosystems in question. In addition, the synoptic phytosociological table summarizes the main characteristics of each plant community in a concise format (Table S1). This table included each community and its dominant plant species, providing a useful overview of the vegetation composition in each area. This table was consistent with trends observed in many terrestrial ecosystems, where herbaceous plants were often the most abundant and diverse group (Bhattarai & Vetaas, 2003). The high proportion of herbaceous species may be attributed to their ability to grow quickly and colonize new areas, as well as their relatively short life spans and high reproductive rates (Giménez et al., 2004, Mota, Luz, et al., 2018). The presence of trees and shrubs suggested that the communities studied may also support more long‐lived and structural vegetation, which can have important ecological functions such as carbon sequestration and soil stabilization (Teague & Kreuter, 2020).

### 4.1 Statistical results

The Rényi diversity method was used to emphases the characteristics and distinctions of the three communities studied and compare their diversity levels (Tóthmérész, 1995). The generated curves were an easy way to reduce the complexity of species structures in relevés and compare their level of diversity (Loreau et al., 2002; Tóthmérész, 1995), as a complement to the assessed diversity indices (Species richness, Shannon and Evennes, Table 2). Analogous to Kindt et al. (2006), we evaluated this method as effective because it considers a wider range of diversity measures, rather than relying on a single index like Shannon or Simpson (Loreau et al., 2002). The *S. purpurea* community (A1) had a lower number of species but a higher degree of coverage and distribution of individuals, resulting in a higher diversity profile compared to the other two sites. In the scrub communities surveyed, Molina et al. (2004) emphasized those characterized by *S. purpurea*, growing on rougher substrates and constituting the vegetation at lower relative altitudes and under the unstable substrate. *H. rhamnoides* community (A2) clearly showed on the curve the regional disadvantages in terms of diversity by a fall of the curve below the other two. The *H. rhamnoides* community (A2) had a higher number of species but a lower diversity due to lower coverage and evenness. This may be due to natural conditions (it is often characterized by a delicate balance between water availability and the water needs of the local ecosystem) and the high proportion of invasive species relative to the total species present in the community. It has indeed been hypothesized that invasive species (*Reynoutria sachalinensis*, *Erigeron canadensis*) may greatly contribute to the decline of native species, particularly in specialized stream habitats (Hoddle, 2004; Tabacchi & Tabacchi, 2001). These invasive species, known for their robust growth and adaptability, may outcompete native plants for resources such as sunlight, water, and nutrients. In specialized stream habitats, where ecological niches are often finely tuned and resources are limited, the aggressive characteristics of invasives could potentially lead to a decline in the abundance and diversity of native plant species.

In the context of our study on riparian forests in the semi‐arid region of southwestern Europe, the observed patterns align with findings in the literature. Aguiar et al. (2013) highlighted that this semi‐arid region exhibited the lowest species diversity among near‐natural southwestern European riparian forests. Specifically, in semi‐arid Mediterranean riparian communities, susceptibility to invasive species was noted, with reports suggesting that up to 30% or more of the species in these areas may be non‐native (Stella et al., 2013).

Addressing the ecological environment, Ruan and Li (2002) identified four layers of soil moisture utilization by species such as *H. rhamnoides*, based on root depth. This utilization pattern, ranging from shallow to adjustment layers, contributes to improved soil and water retention, facilitating moisture replenishment in the 1–1.5 m layer (Ci & Liu, 2010).

The *S. alba* community (A3) had the highest number of species and the high diversity, reflecting the most favorable conservation conditions (Table 2). The present study suggested that regional and local factors may have influenced the variation observed in species richness and vegetation cover. Regional factors, such as climate patterns, geological features, and landscape characteristics, likely played a role in shaping the ecological dynamics of the study area. For example, variations in temperature, precipitation, and soil composition across different regions could potentially impact the diversity and distribution of plant species. However, we note that our study did not directly investigate the relationship between environmental conditions and diversity through methods like modeling (Johnson & Almlöf, 2020). The observed differences in plant communities reflect potential environmental influences, but specific determinants, such as microclimatic conditions, were not explicitly explored. Local factors, such as habitat type and microclimatic conditions were also key determinants of species richness and vegetation cover (Michalet et al., 2015). A similarity was also observed between the community of *S. purpurea* (A1) and *S. alba* community (A3) in terms of the dominance of certain species and distribution trends of the vegetation in the areas. The overlapping of the *S. purpurea* community and the *S. alba* community may be attributed to the similar presence of dominant species within the *Salix* communities. However, despite this, the diversity analyses (Table 2, Figure 3) revealed a weak representation of species at the *H. rhamnoides* community site (Figure 5). We observed a small 15% degree of similarity between communities of *S. purpurea* and *H. rhamnoides* communities that could be explained by the distance between them being relatively closer compared to *S. alba* community, located at a very large distance from both *S. purpurea* and *H. rhamnoides* communities in the Romanian Carpathians. The decrease in available nutrients over time is identified as a key factor that promotes the competitiveness of low‐growing deciduous shrubs and heath shrubs in these ecosystems (Osborne & Kovacic, 1993).

We hypothesized that where the *S. purpurea* community riverbed was wider, had a more complex structure, and a greater number of microhabitats, it allowed a greater diversity of species to develop. Conversely, where the *S. alba* community riverbed was narrower and had fewer microhabitats, it limited the number of species that could live there. Thus, we confirmed that some differences in shrub vegetation composition may be related to environmental conditions (Hoddle, 2004). Our results align with previous research, which suggests that the preference of species for a particular habitat indicates that the development of riparian vegetation is highly dependent on local factors, limiting its spread over larger areas (Slezák et al., 2020). Furthermore, our analysis, employing both boxplot and the Kruskal‐Wallis test, brought to light significant diversity differences among the three communities (Figure 4). In a related study conducted in northern Sweden, Andersson et al. (2000) utilized community similarity as a metric to assess the likeness between various plant community types in riparian vegetation. Although our paper's primary focus is on species diversity, which differs from the specific context of the Swedish study, both investigations share a common thread in exploring plant communities and their diversity along rivers. Following findings from various authors, it can be asserted that, in conducive environments, competition among plant species has the potential to result in a decline in species richness (Lord & Lee, 2001; Mittelbach et al., 2001; Sarr et al., 2005). However, since competitive hierarchies are closely tied to unique combinations of growth resources, competition rarely operates uniformly across large regions or even within smaller landscapes with sufficient complexity. Sarr et al. (2005) found that at local scales, competitive controls can strongly limit richness in the most productive areas with favorable climates that have higher average local richness; however, the richest local environments are found in less productive or frequently disturbed areas where competition is controlled. In areas with unfavorable climates, the overall average richness is low, but the richest local environments are found in locally productive or infrequently disturbed sites (Lord & Lee, 2001; Mittelbach et al., 2001).

Area *S. purpurea* (A1) exhibited a robust correlation with axis 2 (coefficient of .79), emphasizing that the variables or plant communities characterizing *S. purpurea* (A1) were prominently represented along this axis. On the contrary, stronger associations with axis F1 are observed for areas *H. rhamnoides* community (A2) and *S. alba* community (A3), with coefficients of .57 and .69, respectively. This suggested that the variables characterizing A2 and *S. alba* community (A3) exert a more pronounced influence along the F1 axis. Integrating the results of the PCA with previous analyses provides a more comprehensive understanding of the relationships between plant communities and areas (Bejarano et al., 2018), highlighting their unique distribution patterns and associations. The specific plant communities—*S. purpurea* (associated with *S. purpurea* community (A1)) and *H. rhamnoides* (associated with *H. rhamnoides* community (A2))—situated in different quadrants of the biplot indicate their dissimilarities in terms of their relationships with the studied variables. The positioning of *S. alba* community (A3) being distinct from *H. rhamnoides* community (A2) further reaffirms the uniqueness of their distribution patterns.

This supported the idea that each area hosts different plant communities that are not only distinctive in their composition but also in their relationships with the variables (Bejarano et al., 2018; Matteodo et al., 2016; Turner et al., 2005). *H. rhamnoides, S. alba*, and *S. purpurea* were identified as species significantly associated with specific characteristic areas. Their prevalence or characteristics were strongly correlated and influence these specific areas. This implies that these species were indicative of or highly sensitive to the conditions in these areas. *Sambucus ebulus, Populus alba*, and *Rubus caesius* were observed to be associated with both *S. purpurea* community and *S. alba* community zones. Other species are also present: *Equisetum arvense, Populus nigra, Urtica dioica, Viburnum lanthanum*. This suggested that these species have characteristics that allow them to thrive and be relevant in the different environmental conditions represented by zones A1 and *S. alba* community. They could be considered versatile species, able to adapt to different conditions.

Species significantly associated with specific zones, such as *H. rhamnoides* (and also *Calamagrostis arundinacea*), *S. alba* (with *Carpinus betulus*), and *S. purpurea* (and *Tanacetum vulgare*), could serve as ecological indicators. Their presence or absence could indicate the health or special conditions of the respective areas. Monitoring these species could provide information about the general ecological status of the regions concerned. Additionally, there are numerous species with a limited presence (as shown in Figure 5).

### 4.2 Ecology and conservation

Raunkiaer's life form classification system is a widely accepted and practical tool for the ecological classification of plant communities, which can be used to investigate evolutionary processes that shape species assemblages and drive species' morphological traits (Kargar et al., 2017; Loidi et al., 2021; Moradi et al., 2009). In our study, we assessed the Raunkiaer life forms of the plant species within the various communities to understand their ecological adaptations. We identified four major Raunkiaer life forms: chamaephytes, hemicryptophytes, geophytes, and therophytes.

According to Table 3, in the first site, *S. purpurea* community, hemicryptophytes make up the majority of the bioform spectrum at 61%. This suggests that the area being studied likely has a high diversity of perennial plants that were able to survive unfavorable conditions such as drought or frost by having buds located underground. Geophytes (plants with buds positioned beneath the soil surface), microphanerophytes, annual therophytes (plants that complete their life cycle within a year), and biennial therophytes (plants that complete their life cycle within 2 years) each represent 7% of the bioform spectrum. This suggested that these groups are present in relatively low numbers compared to hemicryptophytes.

In the second site, *H. rhamnoides* community, the majority, at almost 48%, represents hemicryptophytes, indicating that they were the most abundant species in the bioform spectrum. The other percent of the table is comprised of microphanerophytes and annual therophytes, each around 10%.

In site *S. alba* community, a high proportion of hemicryptophytes was observed, suggesting an adaptation to survive harsh winters and other environmental stressors. Meanwhile, the percentage of therophytes was low, indicating that the *S. alba* community site may not be subject to significant disturbances that would favor annual or biennial plants over perennials. Additionally, the relatively high percentage of phanerophytes (microphanerophytes and megaphanerophytes) suggested a significant presence of woody vegetation, which may be due to factors such as a long history of forest cover or the presence of water resources that support large tree growth.

Chamaephytes are perennial plants with buds located close to the ground, often in the form of woody shrubs or subshrubs. In our study area, chamaephytes were predominantly found in the higher elevations, where harsh environmental conditions, such as extreme cold and strong winds, are common. These plants exhibit an adaptation to withstand these conditions by maintaining their growing points close to the soil surface, reducing exposure to the elements.

Hemicryptophytes are well‐represented in the mid‐elevation zones of our study area. This life form allows them to regrow from protected buds after seasonal changes and disturbances. Their ecological strategy provides resilience to moderate environmental variations.

We found geophytes to be more common in the lower elevation areas with moist and fertile soils. This life form allows these plants to remain dormant during harsh conditions, such as extreme cold or drought, and resprout from underground storage structures when conditions are favorable.

In our study, therophytes were widespread across all elevations, particularly in disturbed or open areas. Their strategy involves rapid reproduction, with seeds being the primary means of survival and propagation. Therophytes are adapted to exploit opportunities for growth and reproduction when conditions briefly favor their development.

This Raunkiaer life form classification provides insights into how plant species in different communities adapt to environmental challenges and variations across elevations. These differences were essential for understanding the ecological dynamics and plant community composition in our study area, shedding light on how these communities respond to various environmental pressures and disturbances. In addition, hemicryptophytes were the dominant plant type in all three communities studied, which is consistent with similar studies conducted in cold temperate regions and mountainous climates (Bhattarai & Vetaas, 2003; Loidi et al., 2021). The number of chamaephytes was relatively low, with the highest deviation from the typical range observed in these communities. However, chamaephytes were more common in high montane areas, as observed in previous studies (Bhattarai & Vetaas, 2003; Giménez et al., 2004, Mota, Luz, et al., 2018). This decline in chamaephytes could be attributed to climate change (Loidi et al., 2021). The prevalence of geophytes has slightly increased due to dry conditions in some areas of the Carpathians. Therophytes were the next most abundant plant type, showing a minor increase following the typical spectrum. However, there appears to be a weak correlation between therophyte abundance and environmental factors, suggesting that continued anthropogenic (Slezák et al., 2020; Thiel‐Egenter et al., 2009), and overgrazing impacts in the area could have a significant negative impact on the ecosystem.

Other research has shown that therophyte species are more widespread in areas under anthropogenic pressure. Kargar‐Chigani et al. (2017) suggested that the increase in therophytes could be the result of both environmental factors and various human activities such as uncontrolled tourism, timber harvesting, and settlements. Furthermore, an increase in therophytes may lead to changes in moisture levels and water scarcity, as observed by Moradi et al. (2009). The allocation of species life forms differed at different altitudes, with chamaephytes and hemicryptophytes being the most abundant groups at higher altitudes and therophytes showing the highest species richness at lower altitudes. In addition, the biological spectrum of this flora varied from other areas of the Mediterranean basin, with chamaephytes and hemicryptophytes dominating as life forms. Soil was found to be a significant factor in community change, with different soil properties influencing species distribution (Salinas & Casas, 2007). The amplified anthropogenic influence on montane vegetation may be related to the intensification of land use close to human settlements. At all three study sites, we observed a significant proportion of shrubs. Loidi et al. (2021) have suggested that shrubs with similar functions tend to occupy nearby niches, similar to tree species. Functional groups that inhabit wide or transitional environments tend to have wider niches, whereas those specialized in localized habitats tend to have narrower niches. By analyzing the distribution and niche characteristics of shrub species in complex environmental gradients, we can better understand potential conservation targets and threats associated with future land use change and climate change. One theory proposes that deciduous shrubs were more adaptable than trees during the Pleistocene and may have replaced forest communities (Olthoff et al., 2021). Many of these shrubs were originally external to forests and survived in the shade due to seed input from birds. They are sustained by a constant influx of propagules and are composed of individuals with low vitality. Furthermore, many shrub species now exist outside of forests. The difference in average temperature between the present climate and the last ice age has had a greater effect on shrubs than on trees. These findings highlight the importance of plant community diversity in maintaining ecosystem health and function. In addition, the presence of several gymnosperm families suggests that these ecosystems may be important refugia for these ancient plant families. The information obtained in this way may be useful for understanding ecological characteristics and biodiversity in the area and may be important for conservation and management efforts.

This study emphasized that biodiversity can help mitigate negative effects (Loreau et al., 2002).

Human activities such as tourism, agriculture, and sand and gravel extraction threaten riparian plant communities, leading to the introduction of invasive species and habitat fragmentation. In addition, climate change is a significant and pervasive threat (Naiman & Décamps, 1997). Effective riparian management should prioritize biodiversity enhancement within native plant communities (Baxter et al., 2005; Cummins, 1974; Das et al., 2017; Ohmart, 1996). This involves assessing and monitoring habitat conditions (Belsoy et al., 2012; Charles & Dukes, 2008; Koehnken et al., 2020; Peres et al., 2016; Piman et al., 2016). In particular, climate change poses a substantial threat to habitats and species diversity in the Carpathians (Cheval et al., 2014).

Species richness serves as an important metric in conservation studies, providing information on the sensitivity of ecosystems and species (Dala‐Corte et al., 2020). Assessing and monitoring the conservation status of habitats emerge as critical steps in nature protection (Broadmeadow & Nisbet, 2004; Rodewald & Bakermans, 2006). Therefore, riparian management should be dedicated to increasing the biodiversity of native plant communities (Tabacchi & Tabacchi, 2001).

The Natura 2000 network, which serves as the European Union's ecological framework, is designed to support the long‐term conservation of Europe's most endangered species and habitats, effectively limiting biodiversity loss. To protect riparian areas and their biodiversity, many communities have been included in initiatives such as Natura 2000 and the Habitats Directive (www.eea.europa.eu).

We noticed two of the communities in this study were protected under the Natura 2000 program. The *H. rhamnoides* community is located in habitat 2160 Dune with *Hippophaë rhamnoides*, which requires grazing to maintain habitat heterogeneity and species diversity and to prevent secondary succession (Halada et al., 2011). *Hippophaë rhamnoides* dunes (2160) show variable levels of invasion and a medium frequency of exotic species (Fagaras, 2012; Houston, 2020; Leten et al., 2005; Perzanowska et al., 2019).

The *S. alba* community creates galleries within the 92A0 *Salix alba* and *Populus alba* habitat. Findings from a study in central Italy (Carli et al., 2016) support observations of altered floristic structures, where certain exotic plant species—specifically *Robinia pseudoacacia, Amorpha fruticosa*, and *Erigeron canadensis*—have extensively spread along rivers. Effective riparian forest management involves actions that maintain forest stratification, age diversity, and tree species richness, ensuring conservation status and facilitating the restoration of the 92A0 habitat (Carli et al., 2016; Meireles et al., 2009).

In Romania, following the Habitats Directive (EEA, 2021), these communities have been identified with high conservation value, indicating the significance of these habitats in the local context (Doniţă et al., 2005). They are still well represented in the region.

In the Carpathians, as in mountain systems worldwide, protected areas aim to preserve both the natural and cultural values of this unique landscape (Rodewald & Bakermans, 2006). Our study indicates the high conservation status of the three riparian habitats we examined, primarily due to their significant ecological functions.

These habitats are integral to maintaining the structure and functions of the entire landscape(González et al., 2017; Graziano et al., 2022; Janssen et al., 2016), making their preservation imperative (Paine & Ribic, 2002). To achieve this, conserving biodiversity and reducing human pressures on these areas is essential (Fattorini., 2022). Strategies should include preventing habitat fragmentation in the Carpathians and ensuring their inclusion in conservation initiatives such as Natura 2000 and the Habitats Directive (Mandžukovski et al., 2022).

Overall, riparian conservation can meet human needs while providing a network of connected habitats (Allen et al., 2018; Fremier et al., 2015).

## 5 CONCLUSIONS

This study highlights the importance of considering both the number of species and their relative abundance when assessing the diversity of a community. In addition, current findings provide important information for the conservation and management of riverbed communities, highlighting the need to focus on preserving the best conservation conditions and managing invasive species. Moreover, results suggest that to maintain or increase the diversity of riverbed communities (Johnson & Almlöf, 2020), it is necessary to protect the physical characteristics of the riverbeds and the microhabitats that they provide but future studies are needed to support this hypothesis. To sum up, the research provides new insights highlighting the need to preserve the important values of riparian habitats that apply not only locally or regionally but also globally, as riparian vegetation, even if not considered a species of community interest, possesses specific features that make their preservation necessary.

Forwards, there are many areas of future research in the field of riparian woody plant communities in the Romanian Carpathians, as functional diversity, the impact of human activities, climate change or their restoration, and further research in this area will be helpful for the conservation and management of these important ecosystems.

## AUTHOR CONTRIBUTIONS

**Claudia Bita‐Nicolae:** Conceptualization (lead); data curation (equal); investigation (lead); supervision (equal); validation (equal); visualization (equal); writing – original draft (equal); writing – review and editing (equal). **Larisa I. Florescu:** Software (lead); writing – original draft (equal); writing – review and editing (equal). **Dorina Purice:** Data curation (equal); formal analysis (equal); writing – original draft (equal). **Ozkan Kaya:** Supervision (equal); validation (equal); visualization (equal); writing – review and editing (equal).

## CONFLICT OF INTEREST STATEMENT

The authors declare no conflict of interest.

## Supporting information


Table S1

## Data Availability

Data and analysis code are available on Dryad: https://datadryad.org/stash/share/Cd_hRNbIsplQBmXZ_f7s8FVDg‐vCG5WuMVV6_M98KFY.

## References

[ece311361-bib-0001] Aguiar, F. C. , Cerdeira, J. O. , Martins, M. J. , & Ferreira, M. T. (2013). Riparian forests of Southwest Europe: Are functional trait and species composition assemblages constrained by environment? Journal of Vegetation Science, 24(4), 628–638.

[ece311361-bib-0002] Allen, D. C. , Wynn‐Thompson, T. M. , Kopp, D. A. , & Cardinale, B. J. (2018). Riparian plant biodiversity reduces stream channel migration rates in three rivers in Michigan, USA. Ecohydrology, 11(4), e1972.

[ece311361-bib-0003] Andersson, E. , Nilsson, C. , & Johansson, M. E. (2000). Plant dispersal in boreal rivers and its relation to the diversity of riparian flora. Journal of Biogeography, 27(5), 1095–1106.

[ece311361-bib-0004] Bălteanu, D. (2012). Major geomorphological units. In D. Lóczy , M. Stankoviansky , & A. Kotarba (Eds.), Recent landform evolution. The Carpatho‐Balkan‐Dinaric Region. Springer Geography (pp. 250–252). Springer.

[ece311361-bib-0006] Baxter, C. V. , Fausch, K. D. , & Saunders, W. C. (2005). Tangled webs: Reciprocal flows of invertebrate prey link streams and riparian zones. Freshwater Biology, 50(2), 201–220.

[ece311361-bib-0007] Bejarano, M. D. , Nilsson, C. , & Aguiar, F. C. (2018). Riparian plant guilds become simpler and most likely fewer following flow regulation. Journal of Applied Ecology, 55(1), 365–376.

[ece311361-bib-0008] Beldie, A. (1967). Flora and vegetation of the Bucegi Mountains. Academiei.

[ece311361-bib-0009] Belsoy, J. , Korir, J. , & Yego, J. (2012). Environmental impacts of tourism in protected areas. Journal of Environment and Earth Science, 2(10), 64–73.

[ece311361-bib-0010] Bennett, A. F. , Nimmo, D. G. , & Radford, J. Q. (2014). Riparian vegetation has disproportionate benefits for landscape ‐scale conservation of woodland birds in highly modified environments. Journal of Applied Ecology, 51, 514–523. 10.1111/1365-2664.12200

[ece311361-bib-0011] Beschta, R. L. , & Ripple, W. J. (2012). The role of large predators in maintaining riparian plant communities and river morphology. Geomorphology, 157, 88–98.

[ece311361-bib-0501] Bhattarai, K. R. , & Vetaas, O. R. (2003). Variation in plant species richness of different life forms along a subtropical elevation gradient in the Himalayas, east Nepal. Global Ecology and Biogeography, 12(4), 327–340.

[ece311361-bib-0503] Bita‐Nicolae, C. (2022). Distribution and conservation status of the Mountain Wetlands in the Romanian Carpathians. Sustainability, 14(24), 16672.

[ece311361-bib-0504] Bita‐Nicolae, C. (2023). Distribution of the riparian Salix communities in and around Romanian Carpathians. Diversity, 15(3), 397.

[ece311361-bib-0012] Biță‐Nicolae, C. , & Sanda, V. (2011). Cormophlora of Romania. Spontaneous and cultivated cormophytes in Romania. Lambert Academic Publishing.

[ece311361-bib-0013] Biţă‐Nicolae, C. D. (2011). The natural priority habitats in the alpine zone of Bucegi Massif (Romanian southern Carpathians). Botanica Serbica, 35(2), 79–85.

[ece311361-bib-0014] Braun‐Blanquet, J. (1964). Pflanzensoziologie: Grundzüge der Vegetationskunde (3rd ed.). Springer.

[ece311361-bib-0015] Broadmeadow, S. , & Nisbet, T. R. (2004). The effects of riparian forest management on the freshater environment: A literature review of best management practice. Hydrology and Earth System Sciences, 8(3), 286–305.

[ece311361-bib-0016] Butterfield, B. J. , Palmquist, E. C. , & Hultine, K. R. (2021). Regional coordination between riparian dependence and atmospheric demand in willows (*Salix* L.) of western North America. Diversity and Distributions, 27, 377–388. 10.1111/ddi.13192

[ece311361-bib-0017] Capon, S. J. , & Pettit, N. E. (2018). Turquoise is the new green: Restoring and enhancing riparian function in the Anthropocene. Ecological Management & Restoration, 19, 44–53.

[ece311361-bib-0018] Carli, E. , D'Alessandro, E. , Di Marzio, P. , Giancola, C. , Paura, B. , Salerno, G. , & Blasi, C. (2016). Monitoring Natura 2000 habitats: Habitat 92A0 in central Italy as an example. Biogeographia–The Journal of Integrative Biogeography, 31(1), 7–25.

[ece311361-bib-0019] Cavender‐Bares, J. , Kitajima, K. , & Bazzaz, F. A. (2004). Multiple trait associations in relation to habitat differentiation among 17 Floridian oak species. Ecological Monographs, 74, 635–662. 10.1890/03-4007

[ece311361-bib-0020] Chao, A. , & Jost, L. (2015). Estimating diversity and entropy profiles via discovery rates of new species. Methods in Ecology and Evolution, 6(8), 873–882.

[ece311361-bib-0021] Charles, H. , & Dukes, J. S. (2008). Impacts of invasive species on ecosystem services. In Biological invasions (pp. 217–237). Springer.

[ece311361-bib-0022] Cheval, S. , Bîrsan, M. V. , & Dumitrescu, A. (2014). Climate variability in the Carpathian Mountains Region over 1961–2010. Global and Planetary Change, 118, 85–96.

[ece311361-bib-0505] Ci, L. , & Liu, Y. (2010). Biological and technical approaches to control windy desertification. In L. Ci & X. Yang (Eds.), Desertification and its control in China (pp. 351–426). Springer.

[ece311361-bib-0023] Cummins, K. W. (1974). Structure and function of stream ecosystems. Bioscience, 24, 631–641.

[ece311361-bib-0024] Dala‐Corte, R. B. , Melo, A. S. , & Siqueira, T. (2020). Thresholds of freshwater biodiversity in response to riparian vegetation loss in the Neotropical region. Journal of Applied Ecology, 57(7), 1391–1402.

[ece311361-bib-0506] Das, S. , Chou, M. L. , Jean, J. S. , Yang, H. J. , & Kim, P. J. (2017). Arsenic‐enrichment enhanced root exudates and altered rhizosphere microbial communities and activities in hyperaccumulator *Pteris vittata* . Journal of Hazardous Materials, 325, 279–287. 27940117 10.1016/j.jhazmat.2016.12.006

[ece311361-bib-0026] Décamps, H. (1996). The renewal of floodplain forests along rivers: A landscape perspective. Verhandlungen des Internationalen Verein Limnologie, 26, 35–59.

[ece311361-bib-0507] Di Biase, L. , Pace, L. , Mantoni, C. , & Fattorini, S. (2021). Variations in plant richness, biogeographical composition, and life forms along an elevational gradient in a Mediterranean mountain. Plants, 10(10), 2090. 34685898 10.3390/plants10102090 PMC8539301

[ece311361-bib-0027] Dinno, A. (2015). Nonparametric pairwise multiple comparisons in independent groups using Dunn's test. The Stata Journal, 15(1), 292–300.

[ece311361-bib-0028] Doniţă, N. , Paucă‐Comănescu, M. , Popescu, A. , Mihăilescu, S. , & Biriş, I. A. (2005). Habitatele din România. EdituraTehnică Silvică.

[ece311361-bib-0508] Douda, J. , Boublík, K. , Slezák, M. , Biurrun, I. , Nociar, J. , Havrdová, A. , Doudová, J. , Aćić, S. , Brisse, H. , Brunet, J. , Chytrý, M. , Claessens, H. , Csiky, J. , Didukh, Y. , Dimopoulos, P. , Dullinger, S. , FitzPatrick, Ú. , Guisan, A. , Horchler, P. J. , … Zimmermann, N. E. (2016). Vegetation classification and biogeography of European floodplain forests and alder carrs. Applied Vegetation Science, 19(1), 147–163.

[ece311361-bib-0029] EEA . (2021). Terrestrial habitat classification 2021 including crosswalks . www.eea.europa.eu/data‐and‐maps/data/eunis‐habitat‐classification‐1EUNIS

[ece311361-bib-0030] EuroPlusMed . (2012). Euro+MedPlantBase – The information resource for Euro‐Mediterranean plant diversity . http://ww2.bgbm.org/EuroPlusMed/

[ece311361-bib-0031] Fagaras, M. (2012). Habitats of conservative interest and plant communities in the sandy Black Sea coast area of Romania and Bulgaria. Journal of Environmental Protection and Ecology, 13, 1688.

[ece311361-bib-0032] Fattorini, L. , Cervellini, M. , Franceschi, S. , Di Musciano, M. , Zannini, P. , & Chiarucci, A. (2022). A sampling strategy for assessing habitat coverage at a broad spatial scale. Ecological Indicators, 143, 109352.

[ece311361-bib-0033] Ferreira, M. T. , Aguiar, F. C. , & Nogueira, C. (2005). Changes in riparian woods over space and time: Influence of environment and land use. Forest Ecology and Management, 212(1–3), 145–159.

[ece311361-bib-0034] Fierro, P. , Bertrán, C. , Tapia, J. , Hauenstein, E. , Peña‐Cortés, F. , Vergara, C. , Cerna, C. , & Vargas‐Chacoff, L. (2017). Effects of local land‐use on riparian vegetation, water quality, and the functional organization of macroinvertebrate assemblages. Science of the Total Environment, 609, 724–734. 10.1016/j.scitotenv.2017.07.197 28763669

[ece311361-bib-0035] Florescu, L. I. , Moldoveanu, M. , Catană, R. D. , Păceșilă, I. , Dumitrache, A. , Gavrilidis, A. A. , & Iojă, C. I. (2022). Assessing the effects of phytoplankton structure on zooplankton communities in different types of Urban Lakes. Diversity, 14(3), 231.

[ece311361-bib-0036] Fonseca, A. , Zina, V. , Duarte, G. , Aguiar, F. C. , Rodríguez‐González, P. M. , Ferreira, M. T. , & Fernandes, M. R. (2021). Riparian ecological infrastructures: Potential for biodiversity‐related ecosystem Services in Mediterranean human‐dominated landscapes. Sustainability, 13(19), 10508.

[ece311361-bib-0037] Forio, M. A. E. , De Troyer, N. , Lock, K. , Witing, F. , Baert, L. , De Saeyer, N. , Rîșnoveanu, G. , Popescu, C. , Burdon, F. J. , Kupilas, B. , Friberg, N. , Boets, P. , Volk, M. , McKie, B. G. , & Goethals, P. (2020). Small patches of riparian woody vegetation enhance biodiversity of invertebrates. Water, 12, 3070. 10.3390/w12113070

[ece311361-bib-0038] Fremier, A. K. , Kiparsky, M. , Gmur, S. , Aycrigg, J. , Craig, R. K. , Svancara, L. K. , Goble, D. D. , Cosens, B. , Davis, F. W. , & Scott, J. M. (2015). A riparian conservation network for ecological resilience. Biological Conservation, 191, 29–37.

[ece311361-bib-0039] Gałka, M. , Tantau, I. , Carter, V. A. , & Feurdean, A. (2020). The Holocene dynamics of moss communities in subalpine wetland ecosystems in the Eastern Carpathian Mountains, Central Europe. The Bryologist, 123(1), 84–97.

[ece311361-bib-0509] Giménez, E. , Melendo, M. , Valle, F. , Gómez‐Mercado, F. , & Cano, E. (2004). Endemic flora biodiversity in the south of the Iberian Peninsula: Altitudinal distribution, life forms and dispersal modes. Biodiversity and Conservation, 13, 2641–2660.

[ece311361-bib-0040] González, E. , Felipe‐Lucia, M. R. , Bourgeois, B. , Boz, B. , Nilsson, C. , Palmer, G. , & Sher, A. A. (2017). Integrative conservation of riparian zones. Biological Conservation, 211, 20–29.

[ece311361-bib-0041] Google Earth . (2022). earth.google.com

[ece311361-bib-0042] Graziano, M. P. , Deguire, A. K. , & Surasinghe, T. D. (2022). Riparian buffers as a critical landscape feature: Insights for riverscape conservation and policy renovations. Diversity, 14(3), 172.

[ece311361-bib-0510] Guarino, C. , Santoro, S. , & De Simone, L. (2008). Assessment of vegetation and naturalness: a study case in Southern Italy. iForest – Biogeosciences and Forestry, 1(3), 114.

[ece311361-bib-0043] Guo, S. , Zhong, S. , & Zhang, A. (2013). Privacy‐preserving Kruskal–Wallis test. Computer Methods and Programs in Biomedicine, 112(1), 135–145. 23871682 10.1016/j.cmpb.2013.05.023

[ece311361-bib-0044] Halada, L. , Evans, D. , Romão, C. , & Petersen, J. E. (2011). Which habitats of European importance depend on agricultural practices? Biodiversity and Conservation, 20, 2365–2378.

[ece311361-bib-0045] Hammer, Ø. (2012). PAST PAleontological STatistics version 2.15 reference manual .

[ece311361-bib-0511] Hammer, Ø. , & Harper, D. A. (2001). Past: paleontological statistics software package for educaton and data anlysis. Palaeontologia Electronica, 4(1), 1.

[ece311361-bib-0046] Hickin, E. J. (1984). Vegetation and river channel dynamics. Canadian Geographer, 12, 111–126.

[ece311361-bib-0512] Hoddle, M. S. (2004). Restoring balance: using exotic species to control invasive exotic species. Conservation Biology, 18(1), 38–49.

[ece311361-bib-0047] Hoppenreijs, J. H. T. , Eckstein, R. L. , & Lind, L. (2022). Pressures on boreal riparian vegetation: A literature review. Frontiers in Ecology and Evolution, 9, 1–19.

[ece311361-bib-0048] Houston, J. (2020). Conservation of dune habitats in the Atlantic biogeographic region: The dune roadmap for knowledge exchange and networking 2016–2020. In *Change, naturalness and people* (p. 36).

[ece311361-bib-0513] Irl, S. D. , Obermeier, A. , Beierkuhnlein, C. , & Steinbauer, M. J. (2020). Climate controls plant life‐form patterns on a high‐elevation oceanic island. Journal of Biogeography, 47(10), 2261–2273.

[ece311361-bib-0049] Iwata, T. , Nakano, S. , & Inoue, M. (2003). Impacts of past riparian deforestation on stream communities in a tropical rain forest in Borneo. Ecological Applications, 13(2), 461–473. 10.1890/1051-0761(2003)013[0461:ioprdo]2.0.co

[ece311361-bib-0050] Janssen, J. A. M. , Rodwell, J. S. , Criado, M. G. , Gubbay, S. , Haynes, T. , Nieto, A. , & Calix, M. (2016). European red list of habitats. Publications Office of the European Union.

[ece311361-bib-0051] Jansson, R. , Nilsson, C. , Dynesius, M. , & Andersson, E. (2000). Effects of river regulation on river‐margin vegetation: A comparison of eight boreal rivers. Ecological Applications, 10(1), 203–224.

[ece311361-bib-0052] Johnson, R. K. , & Almlöf, K. (2020). Adapting boreal streams to climate change: Effects of riparian vegetation on water temperature and biological assemblages. Freshwater Science, 35, 984–997. 10.1086/687837

[ece311361-bib-0514] Kargar, C. H. , Akbarjavadi, S. , Zahedi Amiri, G. , & Jafari, M. (2017). The floristic composition and biological spectrum of vegetation in the Meymeh region of Northern Isfahan province, Iran. Journal of Applied Ecology and Environmental Research, 15, 415–428.

[ece311361-bib-0515] Kargar‐Chigani, H. , Javadi, S. A. , Zahedi‐Amiri, G. , Khajeddin, S. J. , & Jafari, M. (2017). Vegetation composition differentiation and species‐environment relationships in the northern part of Isfahan Province, Iran. Journal of Arid Land, 9, 161–175.

[ece311361-bib-0054] Kindt, R. , Damme, P. V. , & Simons, A. J. (2006). Tree diversity in western Kenya: Using profiles to characterise richness and evenness. In Forest diversity and management (pp. 193–210). Springer.

[ece311361-bib-0055] Koehnken, L. , Rintoul, M. S. , Goichot, M. , Tickner, D. , Loftus, A. C. , & Acreman, M. C. (2020). Impacts of riverine sand mining on freshwater ecosystems: A review of the scientific evidence and guidance for future research. River Research and Applications, 36(3), 362–370.

[ece311361-bib-0056] Leten, M. , Van Nieuwenhuyse, H. , & Herrier, J. L. (2005). Invasive scrub and trees in the coastal dunes of Flanders (Belgium): An overview of management goals, actions and results. In *Proceedings ‘dunes and estuaries’* (pp. 111–128).

[ece311361-bib-0057] Lind, L. , Hasselquista, E. M. , & Hjalmar, L. H. (2019). Towards ecologically functional riparian zones: A meta‐analysis to develop guidelines for protecting ecosystem functions and biodiversity in agricultural landscapes. Journal of Environmental Management, 249, 1–8. 10.1016/j.jenvman.2019.109391 31445372

[ece311361-bib-0516] Loidi, J. , Chytrý, M. , Jiménez‐Alfaro, B. , Alessi, N. , Biurrun, I. , Campos, J. A. , Čarni, A. , Fernández‐Pascual, E. , Font Castell, X. , Gholizadeh, H. , Indreica, A. , Kavgacı, A. , Knollová, I. , Naqinezhad, A. , Novák, P. , Nowak, A. , Škvorc, Ž. , Tsiripidis, I. , Vassilev, K. , & Marcenò, C. (2021). Life‐form diversity across temperate deciduous forests of Western Eurasia: A different story in the understory. Journal of Biogeography, 48, 2932–2945. 10.1111/jbi.14254

[ece311361-bib-0517] Lord, L. A. , & Lee, T. D. (2001). Interactions of local and regional processes: species richness in tussock sedge communities. Ecology, 82(2), 313–318.

[ece311361-bib-0058] Loreau, M ., Naeem, S ., & Inchausti, P . (Eds.). (2002). Biodiversity and ecosystem functioning: Synthesis and perspectives. Oxford University Press on Demand.

[ece311361-bib-0059] Mandžukovski, D. , Čarni, A. , & Cyril, K. S. S. (2022). Methodius University in Skopje, Hans Em faculty of forest sciences. *Landscape Architecture and Environmental Engineering Federico Fernández González, University of Castilla‐La Mancha MOJA KANCELARIJA DOO SKOPJE Milcho PetrushevskiŽeljko Škvorc*, 500.

[ece311361-bib-0060] Matteodo, M. , Ammann, K. , Verrecchia, E. P. , & Vittoz, P. (2016). Snowbeds are more affected than other subalpine–alpine plant communities by climate change in the Swiss Alps. Ecology and Evolution, 6(19), 6969–6982. 28725374 10.1002/ece3.2354 PMC5513224

[ece311361-bib-0061] Meireles, C. , Neiva, R. , Passos, I. , Vila‐Viçosa, C. , Paiva‐Ferreira, R. , & Pinto‐Gomes, C. (2009). The management and preservation of communitarian interest habitats in the Natural Park of Serra da Estrela (Portugal). Acta Botanica Gallica, 156(1), 79–88.

[ece311361-bib-0518] Michalet, R. , Maalouf, J. P. , Choler, P. , Clément, B. , Rosebery, D. , Royer, J. M. , Schöb, C. , & Lortie, C. J. (2015). Competition, facilitation and environmental severity shape the relationship between local and regional species richness in plant communities. Ecography, 38(4), 335–345.

[ece311361-bib-0062] Micu, D. M. , Dumitrescu, A. , Cheval, S. , & Bîrsan, M. V. (2016). Climate of the Romanian Carpathians. Springer International Publisher.

[ece311361-bib-0063] Milanović, Đ. , & Stupar, V. (2017). Riparian forest communities along watercourses in the Sutjeska National Park (SE Bosnia and Herzegovina). Glasnik Šumarskog Fakulteta Univerziteta u Banjoj Luci, 26, 95–111.

[ece311361-bib-0519] Mittelbach, G. G. , Steiner, C. F. , Scheiner, S. M. , Gross, K. L. , Reynolds, H. L. , Waide, R. B. , Willig, M. R. , Dodson, S. I. , & Gough, L. (2001). What is the observed relationship between species richness and productivity? Ecology, 82(9), 2381–2396.

[ece311361-bib-0520] Molina, J. A. , Pertinez, C. , Diez, A. , & Casemeiro, M. Á. (2004). Vegetation composition and zonation of a Mediterranean braided river floodplain. Belgian Journal of Botany, 137(2), 140–154.

[ece311361-bib-0521] Moradi, G. H. , & Zahedi, A. G. (2009). Life forms of the plants in Irano‐Tourani region and the situation of this region in the world. Journal of Wood and Forest Science and Technology, 16(3), 77–91.

[ece311361-bib-0522] Mota, G. S. , Luz, G. R. , Mota, N. M. , Coutinho, E. S. , Veloso, M. D. D. M. , Fernandes, G. W. , & Nunes, Y. R. F. (2018). Changes in species composition, vegetation structure, and life forms along an altitudinal gradient of rupestrian grasslands in south‐eastern Brazil. Flora, 238, 32–42.

[ece311361-bib-0523] Mota, N. F. D. O. , Watanabe, M. T. C. , Zappi, D. C. , Hiura, A. L. , Pallos, J. , Viveros, R. S. , … Viana, P. L. (2018). Amazon canga: the unique vegetation of Carajás revealed by the list of seed plants. Rodriguésia, 69, 1435–1488.

[ece311361-bib-0065] Mucina, L. , Bültmann, H. , Dierßen, K. , Theurillat, J. P. , Raus, T. , Čarni, A. , Šumberová, K. , Willner, W. , Dengler, J. , García, R. G. , Chytrý, M. , Hájek, M. , Di Pietro, R. , Iakushenko, D. , Pallas, J. , Daniëls, F. J. A. , Bergmeier, E. , Guerra, A. S. , Ermakov, N. , … Tichý, L. (2016). Vegetation of Europe: Hierarchical floristic classification system of vascular plant, bryophyte, lichen, and algal communities. Applied Vegetation Science, 19, 3–264.

[ece311361-bib-0066] Naiman, R. J. , & Décamps, H. (1997). The ecology of interfaces: Riparian zones. Annual Review of Ecology and Systematics, 28, 621–658.

[ece311361-bib-0067] Naiman, R. J. , Décamps, H. , & Pollock, M. (1993). The role of riparian corridors in maintaining regional biodiversity. Ecological Applications, 3(2), 209–212. 27759328 10.2307/1941822

[ece311361-bib-0524] Neuhäuslová, Z. , Douda, J. , Chytrý, M. , & Chytrý, M. (2013). Poříční vrbové křoviny a vrbovotopolové luhy/Riparian willow scrub and willow‐poplar forests. Vegetace České republiky, 4, 45–72.

[ece311361-bib-0068] Ohmart, R. D. (1996). Historical and present impacts of livestock grazing on fish and wildlife resources in western riparian habitats (pp. 245–279). Rangeland Wildlife. Society for Range Management.

[ece311361-bib-0525] Olthoff, A. E. , Martínez‐Ruiz, C. , & Alday, J. G. (2021). Niche characterization of shrub functional groups along an Atlantic‐Mediterranean gradient. Forests, 12(8), 982.

[ece311361-bib-0069] Osborne, L. L. , & Kovacic, D. A. (1993). Riparian vegetated buffer strips in water‐quality restoration and stream management. Freshwater Biology, 29(2), 243–258.

[ece311361-bib-0070] Paine, L. K. , & Ribic, C. A. (2002). Comparison of riparian plant communities under four land management systems in southwestern Wisconsin. Agriculture, Ecosystems & Environment, 92(1), 93–105.

[ece311361-bib-0071] Peres, C. A. , Fearnside, P. M. , Schneider, M. , & Zuanon, J. A. (2016). Hydropower and the future of Amazonian biodiversity. Biodiversity and Conservation, 25(3), 451–466.

[ece311361-bib-0072] Perzanowska, J. , Korzeniak, J. , & Chmura, D. (2019). Alien species as a potential threat for Natura 2000 habitats: A national survey. PeerJ, 7, e8032. 31737451 10.7717/peerj.8032 PMC6855207

[ece311361-bib-0073] Piman, T. , Cochrane, T. A. , & Arias, M. E. (2016). Effect of proposed large dams on water flows and hydropower production in the Sekong, Sesan and Srepok rivers of the Mekong Basin. River Research and Applications, 32(10), 2095–2108.

[ece311361-bib-0526] Raunkiaer, C. (1934). The life forms of plants and statistical plant geography; being the collected papers of C. Raunkiaer. Clarendon Press.

[ece311361-bib-0527] Rivas‐Martinez, S. (1982). Etages bioclimatiques, secteurs chorologiques et séries de végétation de l'Espagne méditerranéenne. Ecologia Mediterranea, 8(1), 275–288.

[ece311361-bib-0074] Rodewald, A. D. , & Bakermans, M. H. (2006). What is the appropriate paradigm for riparian forest conservation? Biological Conservation, 128(2), 193–200.

[ece311361-bib-0075] Rodwell, J. S. , Evans, D. , & Schaminée, J. H. (2018). Phytosociological relationships in European Union policy‐related habitat classifications. RendicontiLincei. ScienzeFisiche e Naturali, 29(2), 237–249.

[ece311361-bib-0076] Romanescu, G. , Stoleriu, C. , & Zaharia, C. (2011). Territorial repartition and ecological importance of wetlands in Moldova (Romania). Journal of Environmental Science and Engineering, 5(11), 1435–1444.

[ece311361-bib-0077] Ruan, C. , & Li, D. (2002). Community characteristics of Hippophaë rhamnoides forest and water and nutrient condition of the woodland in Loess Hilly Region. The Journal of Applied Ecology, 13(9), 1061–1064. 12561161

[ece311361-bib-0078] Salinas, M. J. , & Casas, J. J. (2007). Riparian vegetation of two semi‐arid Mediterranean rivers: Basin‐scale responses of woody and herbaceous plants to environmental gradients. Wetlands, 27(4), 831–845.

[ece311361-bib-0528] Sanda, V. , Ollerer, K. , & Burescu, P. (2008). Fitocenozele din Romania, E. Ars Docendi. Universitatea Bucuresti.

[ece311361-bib-0529] Sarr, D. A. , Hibbs, D. E. , & Huston, M. A. (2005). A hierarchical perspective of plant diversity. The Quarterly Review of Biology, 80(2), 187–212. 16075870 10.1086/433058

[ece311361-bib-0530] Schickhoff, U. (2005). The upper timberline in the Himalayas, Hindu Kush and Karakorum: A review of geographical and ecological aspects. In G. Broll & B. Keplin (Eds.), Mountain ecosystems: studies in treeline ecology (pp. 275–354). Springer.

[ece311361-bib-0080] Schmidt, L. , Teels, B. , & Gray, R. (1991). Wetlands and riparian areas: Their protection, conservation, and restoration. The Agriculture and the Environment, 2, 86–911.

[ece311361-bib-0531] Schwabe, A. (1989). Vegetation complexes of flowing‐water habitats and their importance for the differentiation of landscape units. Landscape Ecology, 2, 237–253.

[ece311361-bib-0081] Sienkiewicz, J. , Kloss, M. , & Grzyb, M. (2001). The floodplain forest ecosystems of Poland. In The floodplain forests in Europe (pp. 249–267). Brill.

[ece311361-bib-0532] Silc, U. (2003). Vegetation of the class *Salicetea purpureae* in Dolenjska (SE Slovenia). Fitosociologia, 40(2), 3–27.

[ece311361-bib-0082] Slezák, M. , Jarolímek, I. , Kochjarová, J. , & Hrivnák, R. (2020). Floodplain forest vegetation in the northern part of the Western Carpathians. Biologia, 75, 1789–1799.

[ece311361-bib-0533] Stella, J. C. , Rodriguez‐Gonzalez, P. M. , Dufour, S. , & Bendix, J. (2013). Riparian vegetation research in Mediterranean‐climate regions: common patterns, ecological processes, and considerations for management. Hydrobiologia, 719, 291–315.

[ece311361-bib-0083] Tabacchi, E. , & Tabacchi, A. M. P. (2001). Functional significance of species composition in riparian plant communities. Journal of the American Water Resources Association, 37(6), 1629–1637.

[ece311361-bib-0084] Tánago, M. G. , & Jalón, D. G. D. (2006). Attributes for assessing the environmental quality of riparian zones. Environmental Science, 25, 389–402.

[ece311361-bib-0085] Teague, R. , & Kreuter, U. (2020). Managing grazing to restore soil health, ecosystem function, and ecosystem services. Frontiers in Sustainable Food Systems, 4, 534187.

[ece311361-bib-0086] The Plant List . (2013). The plant list Version 1.1 . http://www.theplantlist.org/

[ece311361-bib-0534] Thiel‐Egenter, C. , Gugerli, F. , Alvarez, N. , Brodbeck, S. , Cieślak, E. , Colli, L. , Englisch, T. , Gaudeul, M. , Gielly, L. , Korbecka, G. , Negrini, R. , Paun, O. , Pellecchia, M. , Rioux, D. , Ronikier, M. , Schönswetter, P. , Schüpfer, F. , Taberlet, P. , Tribsch, A. , … IntraBioDiv Consortium . (2009). Effects of species traits on the genetic diversity of high‐mountain plants: a multi‐species study across the Alps and the Carpathians. Global Ecology and Biogeography, 18(1), 78–87.

[ece311361-bib-0535] Towpasz, K. , & Stachurska‐Swakon, A. (2008). Alder‐ash and willow communities and their diversity in the Pogorze Strzyzowskie foothills [western Carpathians]. Acta Societatis Botanicorum Poloniae, 77(4), 327–338.

[ece311361-bib-0088] Tóthmérész, B. (1995). Comparison of different methods for diversity ordering. Journal of Vegetation Science, 6(2), 283–290.

[ece311361-bib-0089] Triest, L. , De Greef, B. , De Bondt, R. , & Van Slycken, J. (2000). RAPD of controlled crosses and clones from the field suggests that hybrids are rare in the Salix alba–Salix fragilis complex. Heredity, 84(5), 555–563. 10849080 10.1046/j.1365-2540.2000.00712.x

[ece311361-bib-0090] Turner, K. , Lefler, L. , & Freedman, B. (2005). Plant communities of selected urbanized areas of Halifax, Nova Scotia, Canada. Landscape and Urban Planning, 71(2–4), 191–206.

[ece311361-bib-0091] Turnock, D. (2008). The drive for modernization in inter‐war Eastern Europe: Changes in rurality in the Carpathian mountains 1918‐1945. Geographica Pannonica, 12, 12–38.

[ece311361-bib-0092] United Nations Environment Programme . (2007). Division of early warning, & assessment. Carpathians environment outlook, 2007. United Nations Environment Programme.

[ece311361-bib-0093] Vesipa, R. , Camporeale, C. , & Ridolfi, L. (2017). Effect of river flow fluctuations on riparian vegetation dynamics: Processes and models. Advances in Water Resources, 110, 29–50. 10.1016/j.advwatres.2017.09.028

[ece311361-bib-0094] Wafa'a, A. (2022). Floristic diversity and vegetation of the Zakhnuniyah Island, Arabian Gulf, Saudi Arabia. Heliyon, 8(7), e09996. 35879996 10.1016/j.heliyon.2022.e09996 PMC9307451

[ece311361-bib-0536] Wagner, V. , Chytrý, M. , Jiménez‐Alfaro, B. , Pergl, J. , Hennekens, S. , Biurrun, I. , Knollová, I. , Berg, C. , Vassilev, K. , Rodwell, J. S. , Škvorc, Ž. , Jandt, U. , Ewald, J. , Jansen, F. , Tsiripidis, I. , Botta‐Dukát, Z. , Casella, L. , Attorre, F. , Rašomavičius, V. , … Pyšek, P. (2017). Alien plant invasions in European woodlands. Diversity and Distributions, 23(9), 969–981.

[ece311361-bib-0095] XLSTAT pro . (2013). Data analysis and statistical solution for Microsoft Excel. Addinsoft.

[ece311361-bib-0096] Zhang, J. T. , & Chen, T. (2007). Effects of mixed Hippophaë rhamnoides on community and soil in planted forests in the Eastern Loess Plateau, China. Ecological Engineering, 31(2), 115–121.

