# Supplementary material for: Riparian woody plant communities in the Romanian Carpathians: Species diversity and community structure of Salix and Hippophaë communities
Source: Ecol Evol. 2024 May 21;14(5):e11361. doi: 10.1002/ece3.11361 (PMC11106592; doi:10.1002/ece3.11361)
Supplement: Supplementary file 1 — Table S1 [file ECE3-14-e11361-s001.docx]

Appendix Table S1 Synoptic table, Where: frequency class categories: Class V: 81-100%, Class IV: 61-80%, Class III: 41-60%, Class II: 21-40%, Class I: 1-20%.

| Species | A1 | A2 | A3 |
| --- | --- | --- | --- |
| ***Char.ass.*** |  |  |  |
| Salix purpurea | V |  | IV |
| Hippophaë rhamnoides |  | V |  |
| Salix alba |  |  | V |
| ***Salicetalia*** |  |  |  |
| Populus nigra | IV |  | IV |
| Rubus caesius | III |  | V |
| Populus alba | IV | IV | V |
| Clematis vitalba |  | I | IV |
| Carduus crispus |  |  | III |
| Calystegia sepium |  |  | III |
| Salix fragilis |  |  | iv |
| Salix triandra |  |  | III |
| Populus marilandica |  |  | II |
| Vitis sylvestris |  |  | I |
| Cucubalus baccifer |  |  | III |
| Eupatorium cannabinum |  | I | I |
| Iris pseudacorus |  |  | IV |
| Lycopus europaeus |  |  | IV |
| Lysimachia nummularia |  |  | III |
| Lysimachia vulgaris |  |  | I |
| Myosotis scorpioides |  |  | I |
| Persicaria maculosa |  |  | II |
| Saponaria officinalis |  |  | I |
| Stellaria aquatica |  |  | III |
| Humulus lupus |  |  | IV |
| Tamarix ramosissima |  |  | IV |
| Erigeron annuus |  |  | I |
| Glycyrriza echinata |  |  | I |
| Oenothera biennis |  |  | II |
| Ranunculus repens | III |  | III |
| Rumex obtusifolius | I |  | II |
| Sisymbrium loeselii |  |  | III |
| Sisymbrium strictissimum |  |  | I |
| Urtica dioica | IV |  | I |
| ***Alnu-Ulmion*** |  |  |  |
| Alnus glutinosa |  |  | II |
| Frangula alnus |  | I | I |
| Sambucus nigra |  |  | IV |
| Alnus incana |  | I | I |
| Viburnum opulus |  | II | II |
| Fraxinus angustifolia |  | III | II |
| Stachys palustris |  | I |  |
| Equsetum arvense | II |  | II |
| Mentha spicata | III |  |  |
| Solanum dulcamara | V |  | IV |
| Aristolochia clematitis |  |  | III |
| Glechoma hederacea |  |  | V |
| Lythrum salicaria |  |  | I |
| Physalis alkekengi |  |  | I |
| Valeriana officinalis |  |  | I |
| ***Querco-Fagetea*** |  |  |  |
| Evonymus verrucosa | I |  |  |
| Fraxinus excelsior | I | II |  |
| Acer campestre | IV |  | II |
| Crategus monogyna | IV | III | V |
| Fragaria vesca | I | I |  |
| Geranium robertianum | II | I |  |
| Geum urbanum | II |  |  |
| Glechoma hirsute | III |  |  |
| Ligustrum vulgare | II | II | II |
| Lonicera nigra | III | I |  |
| Myosotis sylvatica | I |  |  |
| Rosa canina | IV | I | II |
| Salvia glutinosa | V | I |  |
| Viburnum lanthanum | III | III |  |
| Quercus pedunculiflora |  |  | I |
| Malus sylvestris |  |  | I |
| Ulmus minor |  |  | III |
| Cornus sanguinea |  | V | III |
| Evonymus europaea |  |  | III |
| Symphytum officinale |  |  | III |
| Populus tremula |  | III |  |
| Acer pseudoplatanus |  | IV |  |
| Carpinus betulus |  | III |  |
| Sorbus acuparia ssp.acuparia |  | III |  |
| Tilia cordata |  | I |  |
| ***Variae Syntaxa*** |  |  |  |
| Bromus sterillis | III | I |  |
| Chelidonium majus | IV |  |  |
| Convolvulus arvensis | II |  |  |
| Lapsana communis | III | III |  |
| Sambucus ebulus | IV |  | II |
| Tanacetum vulgare | V | III |  |
| Artemisia abisitum |  | I |  |
| Reseda lutea | II | III |  |
| Euphorbia esula |  | I |  |
| Echium vulgare | III | II |  |
| Chamenerion dodonaei |  | II |  |
| Calamagrostis arudinaceae |  | I |  |
| Carex distans | III |  |  |
| Juncus effusus | III |  |  |
| Poa trivialis | IV | I |  |
| Potentilla anserina | II | II |  |
| Lotus corniculatus | I | V |  |
| Plantago major | II |  |  |
| Salvia verticillate | I |  |  |
| Picea abies |  | I |  |
| Dactilys glomerata | III | IV |  |
| Vicia cracca | I | I |  |
| Betula pendula | I |  |  |
| Arrhenathereteum elatior | IV | II |  |
| Galium mollugo | II | III |  |
| Leucanthemum ircutianum | III | IV |  |
| Veronica camaedrys | II |  |  |
| Barbarea vulgaris | I |  |  |
| Cardaria draba | II |  |  |
| Cirsium arvense | II |  |  |
| Euphorbia cyparissias | II | III |  |
| Euphorbia salicifolia | III |  |  |
| Petasites hybridus | I |  |  |
| Plantago lanceolata | III | I | II |
| Reynoutria sachalinensis | I | I |  |
| Scrophularia umbrosa | II |  |  |
| Silene acaulis | II |  |  |
| Trifolium pratense | II | I | I |
| Trifolium repens | II |  | I |
| Verbascum phlomoides | II | V | I |
| Leontodon hispidus ssp.danubialis | | I |  |
| Abies alba |  | I |  |
| Polygala comosa |  | IV |  |
| Silene vulgaris |  | II |  |
| Plantago media |  | I | III |
| Carlina vulgaris |  | I |  |
| Salvia pratensis |  | I |  |
| Melica nutans |  | I |  |
| Inula ensifolia |  | III |  |
| Erigeron acris |  | II |  |
| Trifolium campestre |  | IV |  |
| Salix caprea |  | I |  |
| Galium album ssp.album |  | I |  |
| Onobrichis vicifiolia |  | I |  |
| Medicago lupulina |  | I |  |
| Coronilla varia |  | I |  |
| Tusilago farfara |  | I | I |
| Polygala major |  | I |  |
| Erigeron canadensis |  | I |  |
| Scrophularia nodosa |  | I |  |
| Cirsium vulgare |  | I |  |
| Pimpinella saxifraga ssp.saxifraga | | I |  |
| Digitalis grandiflora |  | I |  |
| Ulmus laevis |  | I |  |
| Brachypodium sylvaticum |  | I |  |
| Medicago sativa ssp.falcata |  | I |  |
| Knautia arvensis ssp.arvensis |  | I |  |
| Morus alba |  |  | II |
| Morus nigra |  |  | I |
| Agropyron repens |  |  | II |
| Agrostis stolonifera |  |  | III |
| Althea officinalis |  |  | I |
| Anthryscus sylvestris |  |  | II |
| Arctium lappa |  |  | II |
| Atriplex tatarica |  |  | I |
| Asparagus officinalis |  |  | I |
| Ballota nigra |  |  | II |
| Bellis perennis |  |  | III |
| Bidens tripartite |  |  | IV |
| Cannabis sativa |  |  | I |
| Calamagrostis epigeios |  |  | I |
| Carduus acanthoides |  |  | I |
| Carex riparia |  |  | I |
| Cardamine pratensis |  |  | II |
| Cichorium intybus |  |  | III |
| Cynodon dactylon |  |  | I |
| Dipsacus fullonum |  |  | I |
| Galium aparine |  |  | III |
| Linaria vulgaris |  |  | I |
| Oxalis stricta |  | I | I |
| Phragmites australis |  |  | II |
| Verbena officinalis |  |  | II |
| Rorippa sylvestris |  |  | III |
| Scutellaria galericulata |  |  | III |
| Solanum nigrum |  |  | I |
| Poa pratensis |  |  | I |
| Portulaca oleracea |  |  | I |
